# Exosomal Thomsen–Friedenreich Glycoantigen: A New Liquid Biopsy Biomarker for Lung and Breast Cancer Diagnoses

**DOI:** 10.1158/2767-9764.CRC-23-0505

**Published:** 2024-08-06

**Authors:** Chang-Chieh Hsu, Yafei Su, Kate Rittenhouse-Olson, Kristopher M. Attwood, Wilfrido Mojica, Mary E. Reid, Grace K. Dy, Yun Wu

**Affiliations:** 1 Department of Biomedical Engineering, University at Buffalo, The State University of New York, Buffalo, New York.; 2 For-Robin, Inc., Buffalo, New York.; 3 Department of Biostatistics and Bioinformatics, Roswell Park Comprehensive Cancer Center, Buffalo, New York.; 4 Department of Pathology and Anatomical Sciences, University at Buffalo, The State University of New York, Buffalo, New York.; 5 Department of Medicine, Roswell Park Comprehensive Cancer Center, Buffalo, New York.

## Abstract

Exosomes are nanosized extracellular vesicles released by cells to transport biomolecules such as proteins and RNAs for intercellular communication. Exosomes play important roles in cancer development and metastasis; therefore, they have emerged as potential liquid biopsy biomarkers for cancer screening, diagnosis, and management. Many exosome cargos, including proteins, RNAs, and lipids, have been extensively investigated as biomarkers for cancer liquid biopsy. However, carbohydrates, an important type of biomolecule, have not yet been explored for this purpose. In this study, we reported a new exosomal carbohydrate biomarker, α-linked Thomsen–Friedenreich glycoantigen (TF-Ag-α; Galβ1-3GalNAc-α). To translate our discovery into clinical settings, we developed a surface plasmon resonance–based assay which utilized a unique mAb, JAA-F11, with high specificity to measure the levels of exosomal TF-Ag-α in blood. To the best of our knowledge, we are the first to demonstrate that exosomes carry TF-Ag-α. We detected exosomal TF-Ag-α in as low as 10 μL serum samples from patients with cancer, but in contrast, levels were negligible in those from normal controls. With a total of 233 patients with cancer and normal controls, we showed that exosomal TF-Ag-α detected lung cancer (*n* = 60) and breast cancer (*n* = 95) from normal controls (*n* = 78) with ≥95% and ≥97% accuracy, respectively. These results demonstrated that exosomal TF-Ag-α is a potential liquid biopsy biomarker for cancer diagnosis.

**Significance:**

Exosomes or small extracellular vesicles have emerged as potent biomarkers of cancer liquid biopsy. We discovered a new exosomal carbohydrate marker, TF-Ag-α (Galβ1-3GalNAc-α), and showed that exosomal TF-Ag-α detected both lung and breast cancers with >95% accuracy. Our findings demonstrated that exosomal TF-Ag-α is a promising liquid biopsy biomarker for cancer screening and early detection.

## Introduction

Exosomes are small extracellular vesicles released from cells that serve as important messengers to facilitate intercellular communication. Tumor-derived exosomes (TEX) carry various biomolecules, including nucleic acids, proteins, and lipids, from their parental cells (1) and actively participate in tumor initiation, development, and metastasis. Therefore, TEXs, especially their RNA and protein cargos, have become promising liquid biopsy biomarkers for cancer screening and diagnosis. In June 2019, the FDA granted Breakthrough Device designation to the ExoDx Prostate IntelliScore (EPI) test for prostate cancer risk assessment. The EPI test was the first exosome-based liquid biopsy assay that received such a designation. The EPI test detects exosomal ETS-related gene (*ERG*), prostate cancer antigen 3 (*PCA3*), and SAM pointed domain–containing Ets transcription factor (*SPDEF*) mRNAs in urine samples from patients whose prostate specific antigen (PSA) levels are between 2 and 10 ng/mL. EPI with the cut point at 15.6 is used to distinguish low-risk patients from high-risk patients and help avoid unnecessary prostate biopsies. Exosomal *miR*-21 has been reported to be a potential biomarker for many cancers, including lung cancer (2), breast cancer (3), and liver cancer (4). Exosomal epidermal growth factor receptor (EGFR) and programmed death-ligand 1 (PD-L1) have been used in multiple studies for lung cancer diagnosis (5, 6) and prognosis (7). Recent studies also suggest that exosomal human epidermal growth factor receptor 2 (HER2) (8–10), developmental endothelial locus 1 (Del-1) (11, 12), and fibronectin (13) can serve as biomarkers in breast cancer detection.

The clinical applications of many exosome components, including nucleic acids (DNAs, mRNAs, miRNAs, long noncoding RNAs, and circular RNAs), proteins, and lipids, in cancer diagnosis have been extensively studied. However, carbohydrates, an important type of biomolecule, have not yet been investigated. In this study, we have discovered a new exosomal marker that is carbohydrate-based, Thomsen–Friedenreich glycoantigen (TF-Ag-α; Galβ1-3GalNAc-α). We have shown that exosomal TF-Ag-α is highly sensitive and specific for cancer diagnosis. Expression of the Thomsen–Friedenreich antigen (TF-Ag or CD176) on erythrocytes after exposure to bacterial neuraminidase was first described by Thomsen in 1927 and further detailed by Freidenreich (14, 15). TF-Ag is a typically cryptic antigen that is aberrantly “unmasked” or expressed in cancer cells because of incomplete surface glycosylation (16). It is an oncofetal antigen that has limited expression in normal adult tissues (e.g., placenta). There are two types of linkage configurations for TF-Ag: one is linked in α orientation (expressed in cancer cells only) and the other is linked in β orientation (widely expressed in normal cells; refs. 17, 18). TF-Ag-α plays important roles in tumor growth, immune evasion, and metastasis. Studies have shown correlation of immune response to TF-Ag-α with cancer prognosis and survival (19–23).

In our previous study, we developed a unique mAb, JAA-F11, with high specificity to TF-Ag-α but not TF-Ag-β (24). We showed that TF-Ag-α was expressed in 85% of 1,269 tumor tissue samples from patients with lung, breast, prostate, colon, ovarian, and bladder cancers but not in normal tissues (25). Inspired by the promising results, we aim to investigate whether exosomes carry TF-Ag-α and whether exosomal TF-Ag-α can be used as a blood-based biomarker for cancer diagnosis. To answer these questions, we have developed a surface plasmon resonance (SPR)–based liquid biopsy assay that utilizes the JAA-F11 antibody to sensitively detect exosomal TF-Ag-α in as low as 10 μL serum. Using lung cancer and breast cancer as the disease models, significantly higher levels of exosomal TF-Ag-α were observed in serum samples from a total of 155 patients with cancer (*n* = 60 patients with lung cancer and *n* = 95 patients with breast cancer) than those from normal controls (*n* = 78). Exosomal TF-Ag-α detected lung and breast cancers with an accuracy of ≥0.95 and ≥0.97, respectively. These results demonstrated that exosomal TF-Ag-α is a highly sensitive and specific liquid biopsy biomarker for cancer diagnosis.

## Materials and Methods

### Materials

Methyl-(PEG)4-SH (PEG200, MW 224 Da, 26132) was purchased from Sigma-Aldrich (St. Louis, MO). Biotin-PEG1000-SH (Biotin-PEG1000, MW 1,000 Da, PG2-BNTH-1k) was purchased from Nanocs. NeutrAvidin protein (31000), EZ-Link Micro Sulfo-NHS-LC-Biotinylation Kit (21935), and total exosome isolation (from cell culture medium) kit (4478359) were purchased from Thermo Fisher Scientific. JAA-F11 mouse mAbs (anti–TF-Ag-α mAb) were supplied by For-Robin, Inc. Mouse IgG_3_ antibodies (0105-01, RRID: AB_2793898) were purchased from SouthernBiotech. A SYLGARD 184 silicone elastomer kit (PDMS, 2065622) was purchased from Dow Corning.

### Cell culture

A549 human non–small cell lung cancer (NSCLC) cells (RRID: CVCL_0023) and BEAS-2B human normal bronchial epithelial cells (RRID: CVCL_0168) were cultured in RPMI 1640 medium (11875093; Thermo Fisher Scientific) supplemented with 10% v/v FBS (26140079; Thermo Fisher Scientific) and 1% v/v penicillin–streptomycin (PS, 15140122; Thermo Fisher Scientific). A549 and BEAS-2B cells were seeded at 1.0 × 10^6^ and 1.5 × 10^6^ cells/P100 dish, respectively, and subcultured every 2 to 3 days.

DMS-114 human small cell lung cancer (SCLC) cells (RRID: CVCL_1174) were cultured in Waymouth medium (11220035; Thermo Fisher Scientific) supplemented with 15% v/v exosome-depleted FBS and 1% v/v PS. The exosome-depleted FBS was prepared by centrifuging FBS at 100,000 *g* for 19 hours at 4°C. DMS-114 cells were seeded at 1.5 × 10^6^ cells/P100 dish and subcultured every 3 to 4 days.

MCF-10A human nontumorigenic mammary epithelial cells (RRID: CVCL_0598) were cultured in DMEM/F12 (11330-032; Thermo Fisher Scientific) supplemented with 5% v/v horse serum (16050-122; Thermo Fisher Scientific), 20 ng/mL EGF (AF-100-15; PeproTech), 0.5 mg/mL hydrocortisone (H-0888; Sigma-Aldrich), 100 ng/mL cholera toxin (C-8052; Sigma-Aldrich), 10 μg/mL insulin (I-1882; Sigma-Aldrich), and 1% v/v PS. MCF-7 (RRID: CVCL_0031) and MDA-MB-231 (RRID: CVCL_0062) human breast cancer cells were cultured in DMEM (11995-065; Thermo Fisher Scientific) with 10% v/v FBS and 1% v/v PS. MCF-10A cells were seeded at 2 × 10^6^ cells/P100 dish. MDA-MB-231 and MCF-7 cells were seeded at 1.5 × 10^6^ cells/P100 dish. All cells were subcultured every 2 to 3 days.

All cells used in this study were obtained from ATCC. Short tandem repeat profiling was used for cell line authentication. All cell cultures were tested negative for *Mycoplasma* contamination using the MycoFluor Mycoplasma Detection Kit (M7006; Thermo Fisher Scientific). All cells were used within 2 months after thawing.

### Isolation of exosomes from cell culture–conditioned media

A549, BEAS-2B, MCF-10A, MCF-7, and MDA-MB-231 cells were seeded and allowed to reach 80% confluence in P100 dishes. Then the cells were washed with PBS and cultured in media containing no FBS or horse serum for 2 days. The cell culture–conditioned media were collected and centrifuged at 2,500 *g* for 10 minutes, 4,000 *g* for 30 minutes, and 10,000 *g* for 1 hour at 4°C. The total exosome isolation kit was added to the cell culture–conditioned media at a 1:2 volume ratio according to the manufacturer’s protocol. After incubation at 4°C overnight, the mixture was centrifuged at 10,000 *g* for 1 hour at 4°C to pellet the exosomes. Exosome pellets were resuspended in PBS for future applications.

DMS-114 cells were seeded and allowed to reach 80% confluence in P100 dishes in cell culture medium containing exosome-depleted FBS. The cell culture–conditioned media were collected and processed as described above to isolate exosomes for further analyses.

### Characterization of exosomes by nanoparticle tracking analysis

The nanoparticle tracking analysis system (NanoSight, LM10; Malvern Instruments) was used to measure the size, size distribution, and number concentration of cell-derived exosomes. To maintain the accuracy and consistency of the measurements, exosomes were first diluted with PBS until 50–100 nanoparticles can be tracked in the field of view. The setting of measurement parameters was also identical for all measurements. The camera level was set at 14 during the view-capturing process. The detection threshold was set at 6, and the screen gain was set at 8 during the video processing process.

### Human serum samples

De-identified serum samples and clinical data of normal controls, treatment-naïve patients with lung cancer, and treatment-naïve patients with breast cancer were obtained from the Data Bank and BioRepository Shared Resource at Roswell Park Comprehensive Cancer Center. After obtaining written informed consent from the patients, the Data Bank and BioRepository Shared Resource collected high-quality serum samples and associated epidemiologic and clinical data. After the serum samples were transferred to the University at Buffalo on dry ice, they were stored at −80°C. All serum samples were centrifuged at 10,000 *g* at 4°C for 30 minutes to remove debris and then diluted with PBS at a volume ratio of 1:4 before use. Approvals from Institutional Review Boards of Roswell Park Comprehensive Cancer Center and the University at Buffalo were obtained for the use of human serum samples in this study.

### Exosome isolation and preparation of exosome-depleted human serum samples

The total exosome isolation kit (from serum; 4478360; Thermo Fisher Scientific) was used to isolate exosomes from human serum samples and prepare exosome-depleted serum samples. Briefly, the serum was mixed with the total exosome isolation kit (for serum) at a 2:1 volume ratio according to the manufacturer’s protocol. After incubation at 4°C for 30 minutes, the mixture was centrifuged at 10,000 *g* for 10 minutes at room temperature to pellet exosomes. The supernatant, i.e., exosome-depleted serum, was collected. Exosome pellets were resuspended in 1× PBS.

The exoRNeasy Midi kit (77144; QIAGEN) was used to isolate exosomes from human serum samples. Briefly, 100 µL serum was mixed with 100 µL buffer XBP. The mixture was added into the exoEasy spin column and centrifuged at 500 *g* for 1 minute and at 3,000 *g* for another minute to ensure that no liquid was left in the column. Buffer XWP (3.5 mL) was added into the column, and the column was washed by centrifugation at 3,000 *g* for 1 minute. Finally, 100 µL of buffer XE (76214; QIAGEN) was added to the membrane inside the column and incubated for 1 minute. The column was centrifuged at 3,000 *g* for 5 minutes to elute exosomes. The elute was reapplied on the membrane, and the column was centrifuged at 3,000 *g* for another 5 minutes to maximize the recovery of exosomes.

### Fabrication of SPR biochip

A glass slide (12-550-A3; Thermo Fisher Scientific) was sequentially cleaned with acetone, 200 proof ethanol, and deionized water using a 10-minute sonication for each step. A 2-nm Ti layer and a 49-nm Au layer were then deposited on the glass slide surface by electron-beam evaporation (Kurt J. Lesker Company). The deposition rate for Ti was controlled at 0.8 Å/s, and for Au, it was controlled at 1 Å/s. Finally, the Au-coated glass slide and a piece of PDMS sheet with a 6-mm-diameter hole were treated with oxygen plasma and bound together to complete the assembly of the biochip. The 6-mm-diameter hole served as the sample well.

### Surface modification of SPR biochip

The polyethylene glycol (PEG) mixture (10 mmol/L) was prepared by mixing biotin-PEG1000 with PEG200 at a molar ratio of 1:3 in PBS. The Au-coated surface of the biochip was incubated with 100 μL PEG mixture for 1 hour at room temperature. Excess PEG was washed off with PBS. Then, 100 μL of 50 μg/mL NeutrAvidin was added and incubated for 1 hour at room temperature. After unbound NeutrAvidin was washed off with PBS, 100 μL biotinylated mouse JAA-F11 or mouse IgG_3_ antibodies (50 μg/mL in PBS) were added and incubated at 4°C overnight to conjugate antibodies onto the surface of the biochip through the biotin–avidin interaction. After excess antibodies were removed carefully by PBS washing, the biochip was stored at 4°C for future use.

### Detection of exosomal TF-Ag-α by the SPR biosensor

The biochip was placed on the prism of the SPR biosensor. A 647-nm laser (30 mW power density) was shed through the sample well at the SPR angle, and the intensity of reflected laser beam (I) was recorded using a photodetector. To measure the expression of exosomal TF-Ag-α, water and PBS were first applied on the biochip. The SPR signals of water (I_water_) and PBS (I_PBS_) were recorded for 2 minutes each to establish the baseline measurements. Then 50 μL cell-derived exosomes or human serum samples (10 μL serum was diluted with 40 μL PBS) was applied on the biochip and incubated at room temperature for 1 hour to allow the capture of exosomes expressing TF-Ag-α by JAA-F11 antibodies. After washing off unbound exosomes, the SPR signals from exosomes expressing TF-Ag-α (I_exosomal TF-Ag-α_) were recorded for 2 minutes. The biochip modified with the IgG_3_ control antibodies was used to measure the same sample, and the signal was used to remove background noise caused by nonspecific binding. The expression of exosomal TF-Ag-α was calculated using the following equation:

$$\text{Expression of exosomal TF-Ag-α}={\left(\frac{I_{\text{exosomal}\;\text{TF}-\text{Ag}-\alpha }-\;I_{\text{PBS}}}{I_{\text{PBS}}-I_{\text{water}}}\right)}_{\text{JAA}-F11\;\text{antibody}\;\text{modified}\;\text{biochip}}$$ (A)

$$-\;{\left(\frac{I_{\text{exosomal}\;\text{TF}-\text{Ag}-\alpha \;}-I_{\text{PBS}}}{I_{\text{PBS}}-I_{\text{water}}}\right)}_{\text{IgG}3\;\text{control}\;\text{antibody}\;\text{modified}\;\text{biochip}}.$$

The difference between I_PBS_ and I_water_ was used as the normalization factor to minimize the chip-to-chip variation.

### Scanning electron microscopy

After the capture of exosomes expressing TF-Ag-α on the biochip by JAA-F11 antibodies, the unbound exosomes were removed by washing the biochip with PBS three times. Then, the biochip was dried at 4°C and coated with a layer of carbon before scanning electron microscopy (SEM) imaging. Exosomes expressing TF-Ag-α were characterized using a field emission scanning electron microscope (SU-70; Hitachi High Technologies) with an energy-dispersive X-ray spectrometer.

### Statistical data analysis

ROC analysis and standard two-tailed *t* tests were performed using the SPSS software platform (Ver. 26; IBM, Armonk, NY).

Cutoff value determination: Using the training cohort, an exact logistic regression model was used to generate an ROC curve and corresponding cross-validated AUC assessing the ability of exosomal TF-Ag-α to discriminate between normal controls and cancer groups. The Youden index criterion was used to identify the “optimal” cutoff value for differentiating between normal controls and cancer groups. The sensitivity and specificity associated with the cutoff value were estimated using 95% credible regions obtained by the Jeffreys prior method.

Cutoff value testing: Using the test cohort, an ROC curve and corresponding cross-validated AUC were generated based on the observed exosomal TF-Ag-α values. The cutoff value developed using the training cohort was applied, and the sensitivity and specificity were calculated.

### Data availability

The data generated in this study are available upon request from the corresponding authors.

## Results

### Detection of exosomal TF-Ag-α via a compact SPR biosensor

**Figure 1:**
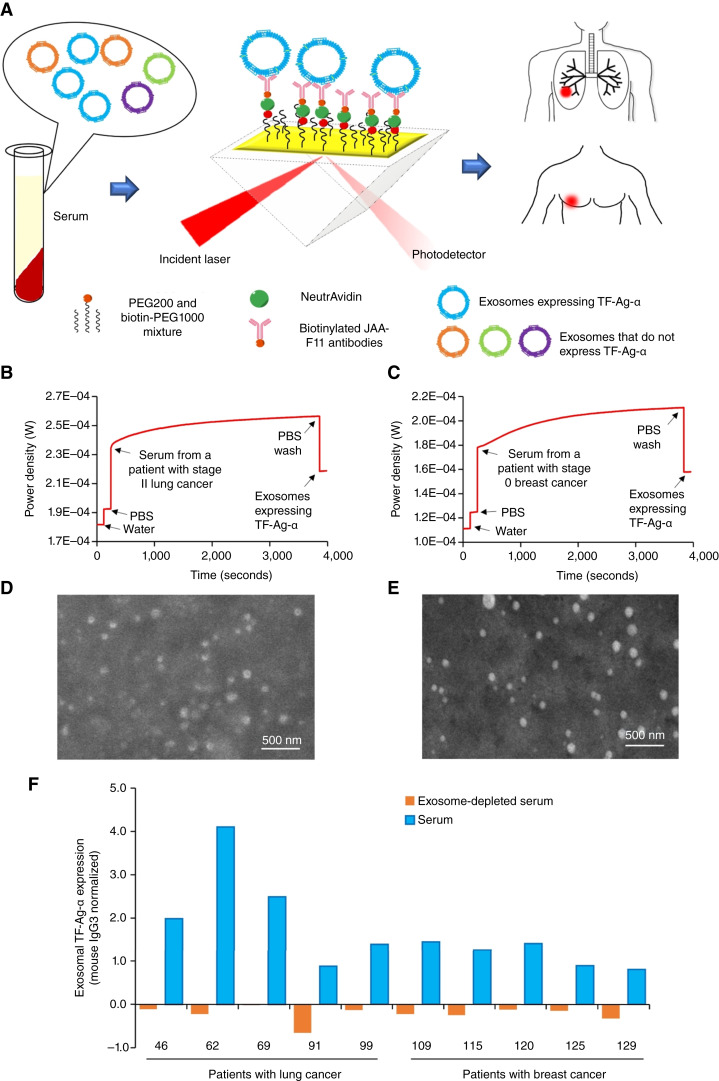
SPR assay detects exosomal TF-Ag-α for cancer diagnosis. **A,** Sensing mechanism of the SPR assay. The assay time was ∼1 hour, and the sample volume was 10 µL serum. Representative SPR curves for the detection of exosomal TF-Ag-α in serum samples from a patient with lung cancer (**B**) and a patient with breast cancer (**C**). SEM images of exosomes expressing TF-Ag-α captured on the biochips from serum samples of a patient with lung cancer (**D**) and a patient with breast cancer (**E**). **F,** Weak signals were detected in exosome-depleted serum samples, whereas strong signals were observed in the same serum samples without exosome depletion (*n* = 10, patient characteristics are provided in Supplementary Tables S1 and S2).

The expression of exosomal TF-Ag-α was measured using the SPR sensing mechanism. As shown in Fig. 1A, the surface of the gold-coated glass slide was modified with a mixture of PEG200 and biotin-PEG1000 [10 mmol/L, 3:1 molar ratio (26, 27)] to provide hydrophilicity and prevent nonspecific binding. Then, NeutrAvidin (50 μg/mL) and biotinylated JAA-F11 antibodies (50 μg/mL) were applied sequentially to attach JAA-F11 antibodies on the surface of the biochip via the avidin–biotin interaction.

A compact SPR biosensor was developed to offer highly sensitive, label-free, and real-time sensing of exosomal TF-Ag-α (6, 27). A polarized laser beam was guided by the prism and reached the biochip that was placed on the top of the prism. The angle of the incident laser was adjusted to trigger SPR on the surface of the gold film, and the intensity of the reflected laser beam was recorded using a photodetector (Fig. 1A). Water and PBS were first applied to collect baseline signals. Then serum samples were added and incubated on the biochip for 1 hour to allow the capture of exosomes expressing TF-Ag-α via JAA-F11 antibodies. The binding of exosomes to JAA-F11 antibodies changed the local refractive index, affected SPR, and increased the intensity of the reflected laser beam. After washing off unbound exosomes, the signals from exosomes expressing TF-Ag-α were recorded. The expression of exosomal TF-Ag-α was calculated using Equation A. Representative SPR curves for the measurements of exosomal TF-Ag-α expression in sera from a patient with stage II lung cancer, a patient with stage 0 breast cancer, and two normal controls are shown in Fig. 1B and C and Supplementary Fig. S1, respectively. The SPR assay was completed in approximately 1 hour and used as low as 10 μL serum.

To confirm that TF-Ag-α is mainly carried by exosomes, SEM was first used to visualize exosomes expressing TF-Ag-α captured on the biochip surface. Individual exosomes were observed as shown in Fig. 1D and E. Next, we used the SPR assay to measure TF-Ag-α levels in exosome-depleted serum samples (*n* = 10, five patients with lung cancer and five patients with breast cancer). Little signal was detected in exosome-depleted serum samples, whereas strong signals were observed in the same serum samples without exosome depletion (Fig. 1F). Finally, we used two exosome isolation kits, total exosome isolation kit (from serum), and exoRNeasy Midi kit, to isolate exosomes from serum samples (*n* = 4, two patients with lung cancer and two patients with breast cancer). The TF-Ag-α expression of isolated exosomes was measured using the SPR assay at the same concentration of 3.29 × 10^11^ exosome/mL. Compared with exosome-depleted serum samples, significantly higher levels of TF-Ag-α were observed in exosomes isolated using both kits (Supplementary Fig. S2). The results from both SEM and SPR assays demonstrated that exosomes carry TF-Ag-α.

### Limit of detection and reproducibility of the SPR assay

The limits of detection (LOD) of the SPR assay were characterized using exosomes derived from A549 NSCLC and MDA-MB-231 breast cancer cells. Cell-derived exosomes (40 μL) were spiked in serum from a normal control (10 μL, H9614; Sigma-Aldrich) and applied on the biochip at various concentrations ranging from 0 (blank control) to 10^11^ exosomes/mL. The blank control was the exosomes isolated from the cell culture medium that went through the same procedure but with no cells. The LOD was calculated as the mean of the blank control plus three times of the SD (28). For A549 NSCLC cell–derived exosomes, the SPR assay detected exosomal TF-Ag-α with the LOD of 5 × 10^9^ exosomes/mL and the linear range from 5 × 10^9^ to 10^11^ exosomes/mL (Supplementary Fig. S3A). For MDA-MB-231 breast cancer cell–derived exosomes, the SPR assay detected exosomal TF-Ag-α with the LOD of 10^9^ exosomes/mL and the linear range from 10^9^ to 10^11^ exosomes/mL (Supplementary Fig. S3B). Our previous results indicated that the exosome concentration in serum is 10^12^ to 10^13^ exosomes/mL (6, 27, 29, 30). Hence, the SPR assay may detect as low as 0.01% exosomes that express TF-Ag-α in total exosomes, demonstrating its high sensitivity.

To determine the repeatability and reproducibility of the SPR assay, the levels of exosomal TF-Ag-α in serum samples from three human subjects were measured by two different operators on three different days, with three replicates per day. The three human subjects consisted of one normal control, one patient with lung cancer, and one patient with breast cancer. As shown in Supplementary Fig. S4, for serum samples from both patients with cancer, the interday coefficient of variation (CV) and intraday CV for operator 1 were ≤18.46% and ≤13.55% and the interday CV and intraday CV for operator 2 were ≤9.27% and ≤5.93%, respectively. For the serum from normal control, because the expression of exosomal TF-Ag-α was below the detection limit, as expected, relatively large CV was observed. The results obtained from the two operators showed a very good correlation with Pearson correlation coefficient of 0.92. These results demonstrated that the SPR assay had excellent repeatability and reproducibility.

### Evaluation of exosomal TF-Ag-α in lung cancer diagnosis

**Figure 2:**
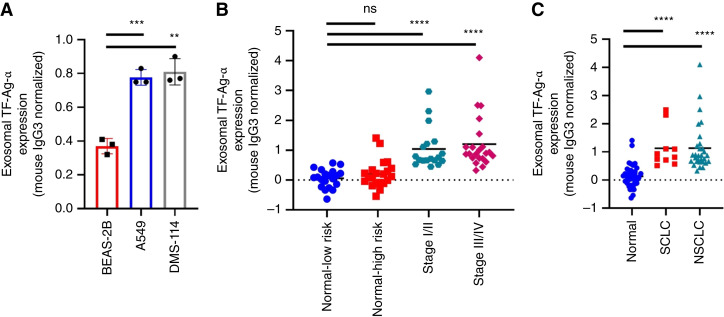
Evaluation of exosomal TF-Ag-α in lung cancer diagnosis. **A,** The expression of TF-Ag-α was significantly higher in exosomes from A549 NSCLC and DMS-114 SCLC cells than those from BEAS-2B normal bronchial epithelial cells at an exosome concentration of 4 × 10^10^ exosomes/mL. **B,** The expression of exosomal TF-Ag-α in serum samples from a training set of patients with lung cancer and normal controls was measured. Exosomal TF-Ag-α expression was significantly higher in serum samples from patients with lung cancer at all stages (*n* = 40; stage I/II: *n* = 18; stage III/IV: *n* = 22) than normal controls (*n* = 41; low risk: *n* = 20; high risk: *n* = 21). **C,** Exosomal TF-Ag-α expression was significantly higher in serum samples from NSCLC (*n* = 30) and SCLC (*n* = 10) than normal controls (*n* = 41). **, *P* < 0.01; ***, *P* < 0.001; ****, *P* < 0.0001; ns, not significant.

The diagnostic value of exosomal TF-Ag-α in detecting lung cancer was first evaluated using cell-derived exosomes. Exosomes derived from A549 NSCLC, DMS-114 SCLC, and BEAS-2B human normal bronchial epithelial cells were applied on the biochips at the same concentration of 4 × 10^10^ exosomes/mL. The expression of TF-Ag-α was 2.1-fold and 2.2-fold higher in exosomes from A549 and DMS-114 cells, respectively, than in those from BEAS-2B cells (Fig. 2A).

Next, the diagnostic performance of exosomal TF-Ag-α was evaluated using serum samples from a training set of patients with lung cancer and matched controls. The training set consisted of 41 normal controls (including 20 patients at low risk of lung cancer and 21 patients at high risk of lung cancer) and 40 patients with lung cancer. Two major subtypes of lung cancer, namely, NSCLC (*n* = 30) and SCLC (*n* = 10), were included. Among them, 18 patients had early-stage (I/II) lung cancer and 22 patients had late-stage (III/IV) lung cancer. The patient characteristics are provided in Table 1 and Supplementary Table S1. The serum sample (10 µL) was diluted with 40 µL PBS and applied on the biochip for the detection of exosomal TF-Ag-α. We observed significantly higher levels of exosomal TF-Ag-α in all lung cancer groups than in normal controls (Fig. 2B and C). Among all normal controls, no significant difference was observed between the low-risk and high-risk groups. We observed slightly higher exosomal TF-Ag-α levels in sera from patients with late-stage (III/IV) lung cancer than patients with early-stage (I/II) lung cancer (Fig. 2B). In addition, there was no significant difference in the expression of exosomal TF-Ag-α between cancer subtypes (Fig. 2C). The lack of significant difference between cancer stages or subtypes may be due to the small sample size, which requires further investigation and validation in large patient cohorts. The other possible reason may be due to the involvement of TF-Ag-α in homotypic aggregation and metastasis. Homotypic aggregation may decrease exosome release of TF-Ag-α, as tumor-associated TF-Ag-α is a critical molecule in initiating tumor/endothelial cell adhesion and facilitating cancer metastasis (31).

**Table 1:** Characteristics of patients with lung cancer

Characteristic	*n* (%)			
	Training set		Test set	
Total patients	81		26	
Normal controls (low risk)	20 (24.7)		6 (23.1)	
Normal controls (high risk)	21 (25.9)		0 (0)	
Cancer	40 (49.4)		20 (76.9)	
	Normal controls (both low-risk and high-risk patients)	Patients with lung cancer	Normal controls (both low-risk and high-risk patients)	Patients with lung cancer
Gender				
Male	24 (29.6)	20 (24.7)	3 (11.5)	10 (38.5)
Female	17 (20.9)	20 (24.7)	3 (11.5)	10 (38.5)
Age (in years)				
Mean	59.9	64.3	62.7	59.7
Median	58	63	62.5	62
Range	42–77	47–78	48–77	41–70
Stage				
I		10 (25.0)		5 (25.0)
II		8 (20.0)		5 (25.0)
III		14 (35.0)		5 (25.0)
IV		8 (20.0)		5 (25.0)
Morphology				
NSCLC		30 (75.0)		14 (70.0)
SCLC		10 (25.0)		6 (30.0)

**Figure 3:**
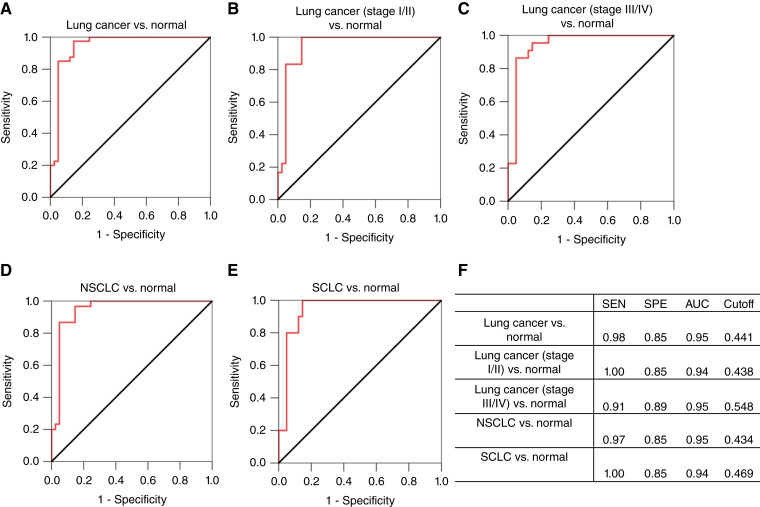
ROC analysis to evaluate diagnostic performance of exosomal TF-Ag-α in lung cancer. ROC curves of comparisons between all patients with lung cancer (*n* = 40) and normal controls (*n* = 41; **A**); early-stage lung cancer (stage I/II, *n* = 18) and normal controls (*n* = 41; **B**); late-stage lung cancer (stage III/IV, *n* = 22) and normal controls (*n* = 41; **C**); NSCLC (*n* = 30) and normal controls (*n* = 41; **D**); and SCLC (*n* = 10) and normal controls (*n* = 41; **E**). Normal controls included 20 low-risk patients and 21 high-risk patients. The sensitivity (SEN), specificity (SPE), AUC, and cutoff values of exosomal TF-Ag-α for each comparison are summarized in **F**.

ROC analysis was used to determine the sensitivity, specificity, and AUC of exosomal TF-Ag-α in lung cancer diagnosis (Fig. 3). Exosomal TF-Ag-α distinguished patients with lung cancer (*n* = 40) from normal controls (*n* = 41) with a sensitivity of 0.98, specificity of 0.85, and AUC of 0.95 at a cutoff value of 0.441 (Fig. 3A and F). To further refine our analysis, we divided patients with lung cancer into early-stage (stage I/II) and late-stage (stage III/IV) groups. Exosomal TF-Ag-α distinguished patients with early-stage lung cancer (*n* = 18) from normal controls (*n* = 41) with a sensitivity of 1.00, specificity of 0.85, and AUC of 0.94 (Fig. 3B and F) and patients with late-stage lung cancer (*n* = 22) from normal controls (*n* = 41) with a sensitivity of 0.91, specificity of 0.95, and AUC of 0.95 (Fig. 3C and F). Patients with lung cancer were also divided based on cancer subtypes, namely, NSCLC and SCLC. Exosomal TF-Ag-α differentiated NSCLC (*n* = 30) from normal controls (*n* = 41) with a sensitivity of 0.97, specificity of 0.85, and AUC of 0.95 (Fig. 3D and F) and SCLC (*n* = 10) from normal controls (*n* = 41) with a sensitivity of 1.00, specificity of 0.85, and AUC of 0.94 (Fig. 3E and F).

**Figure 4:**
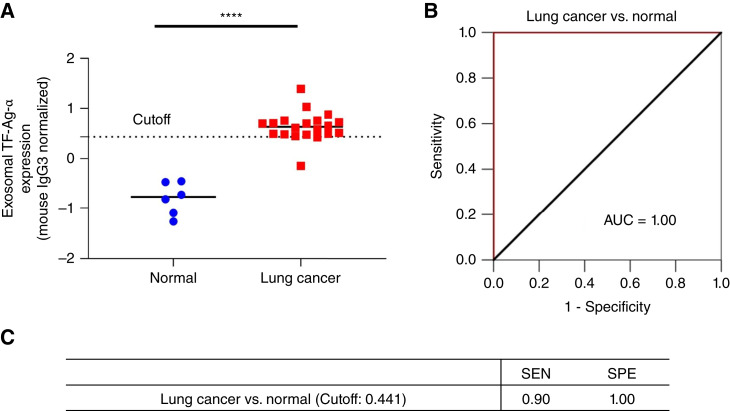
Blinded evaluation of exosomal TF-Ag-α in lung cancer diagnosis using a test set of patients with lung cancer and controls. **A,** Exosomal TF-Ag-α expression was significantly higher in serum samples from patients with lung cancer at all stages (total *n* = 20; stage I/II: *n* = 10; stage III/IV: *n* = 10) than normal controls (*n* = 6; ****, *P* < 0.0001). **B,** ROC curve of comparison between patients with lung cancer (*n* = 20) and normal controls (*n* = 6). The AUC was 1.00. **C,** The sensitivity (SEN) and specificity (SPE) of exosomal TF-Ag-α in distinguishing patients with lung cancer from normal controls using a cutoff value of 0.441 obtained from the training set.

To confirm the diagnostic performance of exosomal TF-Ag-α, we performed a blinded study using a test cohort of patients with lung cancer and controls. The test set consisted of 6 normal controls and 20 patients with lung cancer. Two major subtypes of lung cancer, namely, NSCLC (*n* = 14) and SCLC (*n* = 6), were included. Among them, 10 patients had early-stage (I/II) lung cancer and 10 patients had late-stage (III/IV) lung cancer. The patient characteristics are provided in Table 1 and Supplementary Table S1. The levels of exosomal TF-Ag-α expression were measured by both operator 1 and operator 2. As shown in Fig. 4, exosomal TF-Ag-α expression was significantly higher in serum samples from patients with lung cancer at all stages than normal controls. Based on the observed exosomal TF-Ag-α levels, an ROC curve was plotted, and the AUC was determined to be 1.00. Compared with the training set (AUC = 0.95), exosomal TF-Ag-α showed a higher diagnostic accuracy in the test set (AUC = 1.00). When the cutoff value (0.441) identified from the training set was applied, the sensitivity and specificity of exosomal TF-Ag-α in distinguishing patients with lung cancer from normal controls were 0.90 and 1.00, respectively. These results collectively demonstrated that exosomal TF-Ag-α was a highly sensitive and specific biomarker for the diagnosis of lung cancer.

### Evaluation of exosomal TF-Ag-α in breast cancer diagnosis

**Figure 5:**
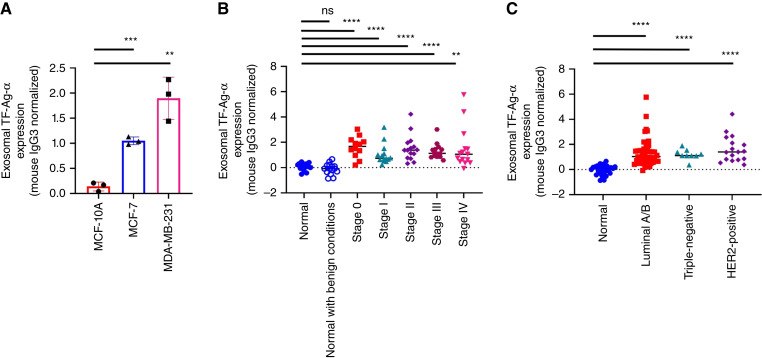
Evaluation of exosomal TF-Ag-α in breast cancer diagnosis. **A,** Expression of TF-Ag-α was significantly higher in exosomes from MDA-MB-231 triple-negative breast cancer and MCF-7 luminal A breast cancer cells than those from MCF-10A normal breast epithelial cells at an exosome concentration of 10^11^ exosomes/mL. **B,** The levels of exosomal TF-Ag-α in serum samples from a training set of patients with breast cancer and normal controls were measured. Exosomal TF-Ag-α expression was significantly higher in serum samples from patients with breast cancer at all stages (*n* = 70; stage 0: *n* = 12; stage I: n = 14; stage II: *n* = 15, stage III: *n* = 14; and stage IV: *n* = 15) than normal controls (*n* = 14) and patients with benign breast conditions (*n* = 15). **C,** Exosomal TF-Ag-α expression was significantly higher in serum samples from patients with luminal A/B (*n* = 44), triple-negative (*n* = 9), and HER2-positive (*n* = 17) breast cancers than normal controls (*n* = 29 including 15 women with benign breast conditions). **, *P* < 0.01; ***, *P* < 0.001; ****, *P* < 0.0001; ns, not significant.

The diagnostic value of exosomal TF-Ag-α in detecting breast cancer was evaluated using both cell-derived exosomes and human serum samples. Exosomes derived from MDA-MB-231, MCF-7 breast cancer, and MCF-10A normal cells were applied on the biochip at the concentration of 10^11^ exosomes/mL. Compared with exosomes from MCF-10A cells, the expression of TF-Ag-α was 13.5-fold higher in exosomes from MDA-MB-231 cells and 7.5-fold higher in exosomes from MCF-7 cells (Fig. 5A).

Next, the levels of exosomal TF-Ag-α in serum samples from a training set of patients were measured using the SPR assay. The training set included 14 normal controls, 16 patients with benign breast conditions, and 70 patients with breast cancer. Benign breast conditions were atypical ductal hyperplasia, atypical lobular hyperplasia, fibroadenoma, fibrocystic change, and intraductal papilloma. All five stages of breast cancer were included (stage 0: *n* = 12; stage I: *n* = 14; stage II: *n* = 15, stage III: *n* = 14; and stage IV: *n* = 15). All major subtypes of breast cancer, including luminal A/B (*n* = 44), triple-negative (*n* = 9), and HER2-positive (*n* = 17), were considered. The patient characteristics are provided in Table 2 and Supplementary Table S2. Similar to lung cancer, we observed significantly higher levels of exosomal TF-Ag-α in all breast cancer groups than normal controls and patients with benign breast conditions. No significant difference was observed between normal controls and patients with benign breast conditions and between cancer stages or subtypes (Fig. 5B and C).

**Table 2:** Characteristics of patients with breast cancer

Characteristic	*n* (%)			
	Training set		Test set	
Total patients	99		30	
Normal controls (no breast conditions)	14 (14.1)		3 (10.0)	
Normal controls (benign breast conditions)	15 (15.2)		2 (6.67)	
Patients with cancer	70 (70.7)		25 (83.3)	
	Normal controls (including patients with benign breast conditions)	Patients with breast cancer	Normal controls (including patients with benign breast conditions)	Patients with breast cancer
Gender				
Male	0 (0)	0 (0)	0 (0)	0 (0)
Female	29 (29.3)	70 (70.7)	5 (16.7)	25 (83.3)
Age (in years)				
Mean	55.9	57.1	56.8	60.8
Median	55	57	50	62
Range	36–77	30–80	47–77	35–78
Stage				
0		12 (12.1)		5 (20.0)
I		14 (14.1)		5 (20.0)
II		15 (15.2)		5 (20.0)
III		14 (14.1)		5 (20.0)
IV		15 (15.2)		5 (20.0)
Morphology				
Luminal A/B		44 (44.4)		19 (76.0)
Triple-negative		9 (9.1)		3 (12.0)
HER2-enriched		17 (17.2)		3 (12.0)

**Figure 6:**
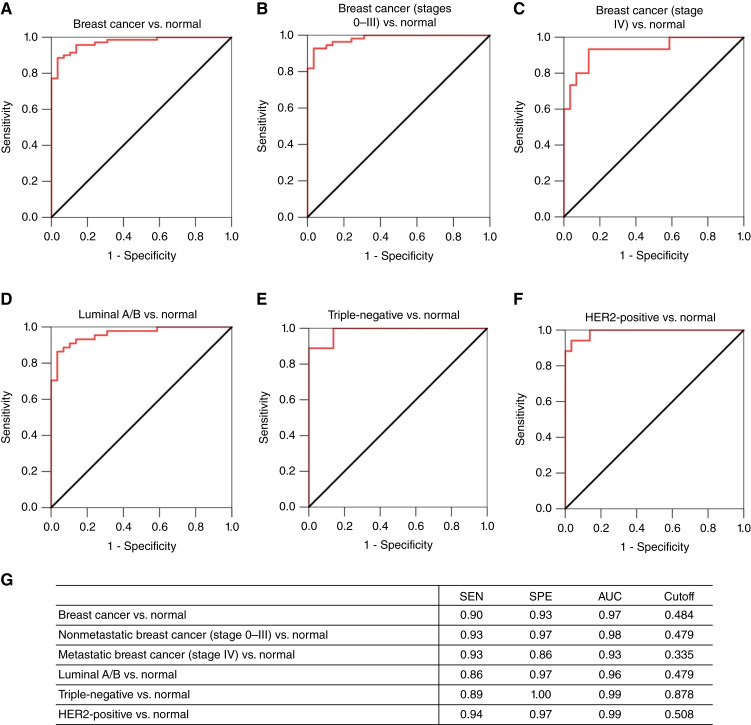
ROC analysis to evaluate diagnostic performance of exosomal TF-Ag-α in breast cancer. ROC curves of comparisons between all patients with breast cancer (*n* = 70) and normal controls (*n* = 29; **A**); nonmetastatic breast cancer (stages 0–III, *n* = 55) and normal controls (*n* = 29; **B**); metastatic breast cancer (stage IV, *n* = 15) and normal controls (*n* = 29; **C**); luminal A/B breast cancer (*n* = 44) and normal controls (*n* = 29; **D**); triple-negative breast cancer (*n* = 9) and normal controls (*n* = 29; **E**); and HER2-positive breast cancer (*n* = 17) and normal controls (*n* = 29; **F**). The sensitivity (SEN), specificity (SPE), AUC, and cutoff values of exosomal TF-Ag-α for each comparison are summarized in **G**.

ROC analysis was performed to determine the sensitivity, specificity, and AUC of exosomal TF-Ag-α in breast cancer diagnosis (Fig. 6). Exosomal TF-Ag-α distinguished patients with breast cancer (*n* = 70) from normal controls (*n* = 29) with a sensitivity of 0.90, specificity of 0.93, and AUC of 0.97 at a cutoff value of 0.484 (Fig. 6A and G). We then divided patients with breast cancer into the nonmetastatic (stages 0–III) and metastatic groups (stage IV). Exosomal TF-Ag-α distinguished patients with nonmetastatic breast cancer (*n* = 55) from normal controls (*n* = 29) with a sensitivity of 0.93, specificity of 0.97, and AUC of 0.98 (Fig. 6B and G) and patients with metastatic breast cancer (*n* = 15) from normal controls (*n* = 29) with a sensitivity of 0.93, specificity of 0.86, and AUC of 0.93 (Fig. 6C and G). For various subtypes of breast cancer, exosomal TF-Ag-α differentiated luminal A/B (*n* = 44) from normal controls (*n* = 29) with a sensitivity of 0.86, specificity of 0.97, and AUC of 0.96 (Fig. 6D and G), triple-negative (*n* = 9) from normal controls (*n* = 29) with a sensitivity of 0.89, specificity of 1.00, and AUC of 0.99 (Fig. 6E and G), and HER2-positive (*n* = 17) from normal controls (*n* = 29) with a sensitivity of 0.94, specificity of 0.97, and AUC of 0.99 (Fig. 6F and G).

**Figure 7:**
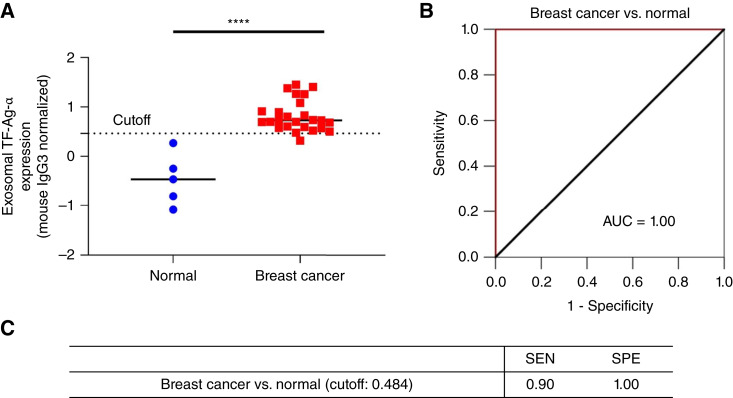
Blinded evaluation of exosomal TF-Ag-α in breast cancer diagnosis using a test set of patients with breast cancer and controls. **A,** Exosomal TF-Ag-α expression was significantly higher in serum samples from patients with breast cancer at all stages (total *n* = 25; stages 0–IV, 5 per stage) than normal controls (*n* = 5 including 2 patients with benign breast conditions; ****, *P* < 0.0001). **B,** ROC curve of comparison between patients with breast cancer (*n* = 25) and normal controls (*n* = 5). The AUC was 1.00. **C,** The sensitivity (SEN) and specificity (SPE) of exosomal TF-Ag-α in distinguishing patients with breast cancer from normal controls using a cutoff value of 0.484 obtained from the training set.

To confirm the diagnostic performance of exosomal TF-Ag-α, we performed a blinded study using a test cohort of patients with breast cancer and controls. The test set consisted of five normal controls (including two patients with benign breast conditions) and 25 patients with breast cancer. All five stages of breast cancer (stages 0–IV, *n* = 5 per stage), and all major subtypes of breast cancer (19 luminal A/B, 3 triple-negative, and 3 HER2-positive) were included. The patient characteristics are provided in Table 2 and Supplementary Table S2. The levels of exosomal TF-Ag-α expression were measured by both operator 1 and operator 2. As shown in Fig. 7, exosomal TF-Ag-α expression was significantly higher in serum samples from patients with breast cancer than normal controls. Based on the observed exosomal TF-Ag-α levels, an ROC curve was plotted, and the AUC was determined to be 1.00. Compared with the training set (AUC = 0.97), exosomal TF-Ag-α showed a higher diagnostic accuracy in the test set (AUC = 1.00). When the cutoff value (0.484) identified from the training set was applied, the sensitivity and specificity of exosomal TF-Ag-α in distinguishing patients with breast cancer from normal controls were 0.92 and 1.00, respectively. All results demonstrated that exosomal TF-Ag-α was a sensitive biomarker for breast cancer diagnosis.

## Discussion

Laboratory tests, medical imaging, and biopsies are routinely used in clinical cancer diagnosis. For example, low-dose chest CT is a common lung cancer screening modality. Mammography and ultrasound serve as the main methods for breast cancer screening. However, high false-positive rate, risk of overdiagnosis, and unnecessary radiation exposure are the main challenges (32–38). Although computer-assisted methods such as deep learning have been developed to assist low-dose chest CT (39) and mammograms (40) and great progress has been made so far, accuracy of these approaches is still poor and subjective. In fact, tissue biopsy is the gold standard in clinics; however, it is an invasive procedure, cannot be easily repeated in certain patients such as those with lung cancer, and is challenged by tumor heterogeneity (41).

Liquid biopsy detects circulating cancer biomarkers in body fluids, such as blood, urine, and saliva. It is a minimal- or noninvasive test, provides supplementary information in the wake of medical imaging or tissue biopsy, and assists with cancer screening, diagnosis, and treatment response monitoring (42, 43). However, current circulating blood-based biomarkers, such as carcinoembryonic antigen (CEA), PSA, cancer antigen 125 (CA125), and CA19-1, suffer from poor sensitivity and specificity. Recent studies have demonstrated that circulating tumor cells (CTC), ctDNAs, and TEXs are promising biomarkers to enable highly sensitive and specific cancer diagnosis. Compared with CTCs and ctDNAs, TEXs are particularly suitable for cancer diagnosis because TEXs exist in much larger quantities than CTCs and ctDNAs and thus may provide a higher diagnostic sensitivity. TEXs are much more stable than CTCs and ctDNAs and do not require immediate or special sample processing, which are necessary attributes for cost-effective, large-scale, routine local or central laboratory–based cancer tests. This stability allows for biomarker discovery/validation using samples in biorepositories. TEXs are secreted by living cancer cells, unlike ctDNA from “dying cells,” thus providing a more precise portrait of tumor status (44).

The discovery of TEX markers represents a new technological approach in cancer liquid biopsy platforms. Many TEX cargos, such as miRNAs and proteins, have been extensively investigated. However, TEX carbohydrate markers have not yet been explored. Our previous study showed that TF-Ag-α expression in tissue was detected by the JAA-F11 antibody in 85% of 1,269 samples of breast, lung, prostate, colon, bladder, and ovarian cancers but not in normal tissues. Specifically, for lung and breast cancers, 84% of 235 lung cancer samples and 88% of 444 breast cancer samples tested positive for TF-Ag-α expression. These results suggest that TF-Ag-α may be a pan-cancer biomarker with high sensitivity and specificity (25). This exciting finding urged us to examine if exosomes carry TF-Ag-α and if exosomal TF-Ag-α could be used as a liquid biopsy biomarker for cancer diagnosis. In this work, we selected lung and breast cancers as the disease models and demonstrated that exosomes carry TF-Ag-α. In order to translate our discovery into clinical settings, we developed a highly sensitive SPR assay to detect exosomal TF-Ag-α directly in serum without sample preparation. The results from the LOD experiments showed that the SPR assay may detect as low as 0.01% exosomes that express TF-Ag-α in total exosomes. The SPR assay also showed excellent intraday, interday, and interoperator reproducibility. The SPR assay embraced a user-friendly design, only requiring basic understanding of the SPR sensing mechanism and experience in using a micropipette to perform this assay. With a total of 233 patients and controls in both training and test sets, exosomal TF-Ag-α distinguished patients with lung cancer (stages I–IV) and patients with breast cancer (stages 0–IV) from normal controls with AUC values ≥0.95 and ≥0.97, respectively. These results collectively demonstrated the great potential of exosomal TF-Ag-α in lung and breast cancer liquid biopsies. Due to its expression across various malignancies, however, additional platforms to establish the tissue type of origin are necessary to confirm the specific cancer diagnosis.

In the future, to facilitate the clinical translation of the exosomal TF-Ag-α–based assay, we will follow Clinical Laboratory Improvement Amendments standards and perform a comprehensive characterization of assay performances including accuracy, precision, reportable range, reference interval, analytical sensitivity and analytical specificity. We will further validate the diagnostic potential of exosomal TF-Ag-α with large validation cohorts of normal controls, other non-cancer disease states, and patients with cancer. We noticed that there were no significant differences in the expression of exosomal TF-Ag-α among different cancer stages and subtypes. This may be due to the small sample size for each cancer stage and subtype in each cancer group. Therefore, future studies with larger sample size will be performed to further investigate the role of exosomal TF-Ag-α in enhancing the positive predictive value of standard cancer screening modalities as a cancer diagnostic and staging biomarker and to further investigate if exosomal TF-Ag-α is a sufficient biomarker for cancer subtyping. We will expand the cancer type to include more cancers such as colon, prostate, and ovarian cancers, as we already showed the high expression of TF-Ag-α in these cancer tissues in our previous study (25). In addition, exosomal TF-Ag-α measurement may potentially be utilized as a noninvasive companion diagnostic platform for cancer-specific assessment of other membrane biomarkers expressed on the cell surface, e.g., to evaluate dynamic changes in HER2 expression status. Lastly, a humanized version of JAA-F11 (hJAA-F11) with high specificity to TF-Ag-α and low immunogenicity has been developed for clinical administration for imaging and therapy (19, 45). The exosomal TF-Ag-α assay developed in this work may also be used as a companion test to select patients for hJAA-F11 therapy and monitor treatment response.

## Supplementary Material

Table S1 Supplementary Table S1. Characteristics of Lung Cancer Patients and Controls.

Table S2 Supplementary Table S2. Characteristics of Breast Cancer Patients and Controls.

Figure S1 Supplementary Figure S1. SPR assay detects exosomal TF-Ag-α for cancer diagnosis. Representative SPR curves for the detection of exosomal TF-Ag-α in serum samples from (a) a Stage II lung cancer patient using a biochip modified with IgG3 negative control antibodies; (b) a Stage 0 breast cancer patient using a biochip modified with IgG3 negative control antibodies, (c) a male normal control using a biochip modified with JAA-F11 antibodies, (d) a male normal control using a biochip modified with IgG3 negative control antibodies, (e) a female normal control using a biochip modified with JAA-F11 antibodies, and (f) a female normal control using a biochip modified with IgG3 negative control antibodies.

Figure S2 Supplementary Figure S2. SPR assay demonstrated exosomes carry TF-Ag-α. Exosomes were isolated from cancer patient serum samples using both total exosome isolation kit (from serum) (ThermoFisher) and exoRNeasy midi kit (Qiagen). Significantly higher levels of TF-Ag-α were observed in exosome samples isolated using both kits than the exosome-depleted serum samples. The exosome concentration was 3.29x10^11 exosomes/mL.

Figure S3 Supplementary Figure S3. Sensing performance of SPR assay in detecting exosomal TF-Ag-α. Exosomes from A549 NSCLC cells (a) and MDA-MB-231 breast cancer cells (b) were spiked in the serum of a normal control at concentrations of 0 to 10^11 exosomes/mL. The expression of exosomal TF-Ag-α was measured using the SPR assay. For A549 cell-derived exosomes, the LOD was 5×10^9 exosomes/mL and the linear range was from 5×10^9 to 10^11 exosomes/mL. For MDA-MB-231 cell-derived exosomes, the LOD was 10^9 exosomes/mL and the linear range was from 10^9 to 10^11 exosomes/mL.

Supplementary Figure S4 Supplementary Figure S4. Repeatability and reproducibility of SPR assay in detecting exosomal TF-Ag-α. The levels of exosomal TF-Ag-α in serum samples from one normal control, one lung cancer patient and one breast cancer patient were measured by operator 1 (a) and operator 2 (b) on 3 different days, 3 replicates per day. The mean, standard deviation, and coefficient of variation (CV) were reported. (c) Pearson correlation of results from operator 1 and operator 2.
