# Supplementary material for: Exosomal Thomsen–Friedenreich Glycoantigen: A New Liquid Biopsy Biomarker for Lung and Breast Cancer Diagnoses
Source: Cancer Res Commun. 2024 Aug 6;4(8):1933–45. doi: 10.1158/2767-9764.CRC-23-0505 (PMC11302018; doi:10.1158/2767-9764.CRC-23-0505)
Supplement: Table S1 — Supplementary Table S1. Characteristics of Lung Cancer Patients and Controls. [file crc-23-0505_table_s1_suppst1.pdf]

Supplementary Table S1. Characteristics of Lung Cancer Patients and Controls

ID	Cancer type	Race	Gender	Age	Stage	Exosomal TF-Ag-α level (A.U.)
**Training set (41 normal controls and 40 cancer cases)**						
1	Normal control (low risk)	White	M	50		0.35
2	Normal control (low risk)	White	F	65		0.28
3	Normal control (low risk)	White	F	56		0.04
4	Normal control (low risk)	White	M	57		0.20
5	Normal control (low risk)	White	F	60		-0.23
6	Normal control (low risk)	White	M	61		0.22
7	Normal control (low risk)	White	M	60		-0.64
8	Normal control (low risk)	White	M	68		0.53
9	Normal control (low risk)	White	M	65		-0.26
10	Normal control (low risk)	Black	M	50		-0.34
11	Normal control (low risk)	White	F	77		0.02
12	Normal control (low risk)	White	M	54		0.08
13	Normal control (low risk)	White	F	70		0.15
14	Normal control (low risk)	White	F	76		0.10
15	Normal control (low risk)	White	F	60		0.16
16	Normal control (low risk)	White	F	64		0.43
17	Normal control (low risk)	White	F	56		-0.14
18	Normal control (low risk)	White	M	49		-0.04
19	Normal control (low risk)	White	M	73		-0.34
20	Normal control (low risk)	White	M	57		0.57
21	Normal control (high risk)	White	M	53		1.41
22	Normal control (high risk)	White	M	42		1.23
23	Normal control (high risk)	White	M	55		0.12
24	Normal control (high risk)	White	F	55		0.61
25	Normal control (high risk)	White	F	55		0.22
26	Normal control (high risk)	White	M	73		-0.08
27	Normal control (high risk)	White	M	52		0.59
28	Normal control (high risk)	White	F	48		-0.54
29	Normal control (high risk)	White	M	62		0.08
30	Normal control (high risk)	White	F	71		0.23
31	Normal control (high risk)	White	F	63		0.29
32	Normal control (high risk)	White	M	55		-0.33
33	Normal control (high risk)	Unknown	M	58		0.20
34	Normal control (high risk)	White	M	57		0.34

35	Normal control (high risk)	White	F	72		-0.19
36	Normal control (high risk)	White	M	69		0.39
37	Normal control (high risk)	White	M	53		-0.11
38	Normal control (high risk)	White	F	51		-0.11
39	Normal control (high risk)	White	F	52		0.07
40	Normal control (high risk)	White	M	65		-0.04
41	Normal control (high risk)	White	M	66		-0.01
42	Squamous cell carcinoma, keratinizing, NOS	White	F	52	1B	0.64
43	Squamous cell carcinoma, NOS	White	M	59	1A	0.66
44	Adenocarcinoma, NOS	White	F	75	1A	0.79
45	Adenocarcinoma, NOS	White	F	56	1A	0.53
46	Adenocarcinoma, NOS	White	M	60	1A	1.99
47	Squamous cell carcinoma, NOS	White	M	76	1A	1.21
48	Adenocarcinoma, NOS	White	M	60	1B	0.89
49	Adenocarcinoma, NOS	White	M	53	1B	2.97
50	Small cell lung cancer	White	F	78	1A2	0.71
51	Small cell lung cancer	White	F	73	1A3	0.51
52	Adenocarcinoma with mixed subtypes	White	F	74	2	0.66
53	Squamous cell carcinoma, NOS	White	M	66	2A	0.65
54	Adenocarcinoma, NOS	White	F	70	2B	0.67
55	Adenocarcinoma, NOS	White	F	61	2B	0.45
56	Adenocarcinoma, NOS	White	M	57	2B	0.76
57	Squamous cell carcinoma, NOS	White	M	65	2B	1.11
58	Adenocarcinoma, NOS	White	M	63	2B	1.29
59	Small cell lung cancer	White	F	70	2B	2.31
60	Mucinous adenocarcinoma	White	M	57	3B	0.87
61	Squamous cell carcinoma, NOS	White	M	54	3A	0.32
62	Adenocarcinoma, NOS	White	F	58	3B	4.10
63	Adenocarcinoma, NOS	White	F	64	3A	0.72
64	Adenocarcinoma, NOS	White	F	55	3B	1.11
65	Squamous cell carcinoma, NOS	White	F	63	3A	2.06
66	Squamous cell carcinoma, NOS	White	M	67	3B	1.01
67	Adenocarcinoma, NOS	White	F	47	3A	0.90
68	Adenocarcinoma, NOS	White	F	77	3A	0.44

69	Small cell lung cancer	White	F	71	3B	2.49
70	Small cell lung cancer	White	F	60	3A	0.81
71	Small cell lung cancer	White	M	61	3B	0.74
72	Small cell lung cancer	White	M	62	3A	0.57
73	Small cell lung cancer	White	M	62	3A	1.10
74	Adenocarcinoma, NOS	White	F	73	4	0.87
75	Adenocarcinoma, NOS	White	F	69	4	2.51
76	Squamous cell carcinoma, NOS	White	M	58	4	1.55
77	Adenocarcinoma, NOS	White	M	62	4A	0.61
78	Squamous cell carcinoma, NOS	White	M	77	4B	0.88
79	Squamous cell carcinoma, NOS	White	F	70	4B	0.73
80	Small cell lung cancer	Black	M	70	4A	0.93
81	Small cell lung cancer	White	M	68	4B	1.11
**Test set (6 normal controls and 20 cancer cases)**						
82	Normal control	White	M	61		-1.26
83	Normal control	White	M	65		-0.72
84	Normal control	White	M	63		-0.45
85	Normal control*	White	F	77		-1.09
86	Normal control*	White	F	62		-0.47
87	Normal control*	White	F	48		-0.81
88	Adenocarcinoma, NOS	White	M	69	1	0.70
89	Squamous cell carcinoma, NOS	White	M	57	1	0.48
90	Adenocarcinoma, NOS	White	F	52	1	0.49
91	Adenocarcinoma, NOS	White	M	64	1	0.88
92	Adenocarcinoma, NOS	White	F	70	1	0.49
93	Adenocarcinoma, NOS	White	M	68	2	0.72
94	Adenocarcinoma, NOS	White	M	48	2	0.76
95	Adenocarcinoma with mixed subtypes	White	M	61	2	0.76
96	Adenocarcinoma with mixed subtypes	White	F	64	2	0.45
97	Squamous cell carcinoma, NOS	White	M	49	2	0.71
98	Adenocarcinoma, NOS	White	F	62	3	0.48
99	Adenocarcinoma, NOS	White	F	55	3B	1.39
100	Small cell carcinoma, NOS	White	M	62	3	1.03
101	Small cell carcinoma, NOS	White	F	62	3	0.58
102	Small cell carcinoma, NOS	White	F	51	3	0.70
103	Adenocarcinoma, NOS	White	F	41	4	-0.15

104	Adenocarcinoma, NOS	White	M	55	4	0.51
105	Small cell carcinoma, NOS	White	F	67	4	0.42
106	Small cell carcinoma, NOS	White	M	66	4	0.61
107	Small cell carcinoma, NOS	White	F	64	4	0.66

*These normal controls are the same normal controls #100, #101 and #102 in Table S2.
