# Supplementary material for: Exosomal Thomsen–Friedenreich Glycoantigen: A New Liquid Biopsy Biomarker for Lung and Breast Cancer Diagnoses
Source: Cancer Res Commun. 2024 Aug 6;4(8):1933–45. doi: 10.1158/2767-9764.CRC-23-0505 (PMC11302018; doi:10.1158/2767-9764.CRC-23-0505)
Supplement: Table S2 — Supplementary Table S2. Characteristics of Breast Cancer Patients and Controls. [file crc-23-0505_table_s2_suppst2.pdf]

Supplementary Table S2. Characteristics of Breast Cancer Patients and Controls

ID	Cancer type	Race	Gender	Age	Stage	Exosomal TF-Ag-α level (A.U.)
**Training set (29 normal controls and 70 cancer cases)**						
1	Normal control	White	F	36		0.01
2	Normal control	White	F	44		0.30
3	Normal control	White	F	48		0.46
4	Normal control	White	F	55		0.30
5	Normal control	White	F	57		0.09
6	Normal control	White	F	69		0.04
7	Normal control	White	F	65		-0.06
8	Normal control	White	F	74		0.40
9	Normal control	White	F	73		-0.29
10	Normal control	White	F	50		-0.52
11	Normal control	White	F	52		-0.42
12	Normal control	White	F	63		0.28
13	Normal control	White	F	56		0.22
14	Normal control	White	F	64		0.15
15	Abnormal mammogram	Black	F	41	Benign	0.08
16	Atypical ductal hyperplasia (ADH)	White	F	77	Benign	0.18
17	Atypical ductal hyperplasia	Black	F	47	Benign	0.66
18	Atypical ductal hyperplasia	White	F	59	Benign	-0.08
19	Atypical lobular hyperplasia (ALH)	White	F	41	Benign	-0.21
20	Atypical lobular hyperplasia	White	F	52	Benign	0.10
21	Atypical lobular hyperplasia	White	F	55	Benign	-0.31
22	Fibroadenoma	White	F	47	Benign	-0.53
23	Fibroadenoma	White	F	50	Benign	-0.20
24	Fibroadenoma	White	F	57	Benign	-0.35
25	Fibrocystic Change	Black	F	53	Benign	-0.86
26	Fibrocystic Change	White	F	56	Benign	-0.83
27	Intraductal Papilloma	White	F	49	Benign	-0.08
28	Intraductal Papilloma	White	F	55	Benign	0.22
29	Intraductal Papilloma	White	F	64	Benign	0.47
30	Ductal carcinoma in situ (DCIS)	White	F	64	0	1.34
31	DCIS	White	F	56	0	1.92
32	DCIS	White	F	65	0	0.21
33	DCIS	White	F	33	0	1.85
34	DCIS	White	F	70	0	3.04

35	DCIS	Asian	F	61	0	0.49
36	DCIS	White	F	44	0	1.51
37	DCIS	White	F	62	0	1.42
38	DCIS	White	F	76	0	2.04
39	Invasive carcinoma, no special type	unknown	F	61	0	0.97
40	Invasive carcinoma, no special type	White	F	54	0	2.57
41	Intraductal carcinoma, noninfiltrating	White	F	48	0	2.19
42	Infiltrating duct carcinoma	White	F	48	1	3.20
43	Infiltrating duct carcinoma	White	F	58	1	0.69
44	Infiltrating duct carcinoma	White	F	57	1	0.71
45	Infiltrating duct carcinoma	White	F	68	1	0.66
46	Infiltrating duct carcinoma	White	F	65	1	0.48
47	Infiltrating duct carcinoma	White	F	77	1	1.11
48	Infiltrating duct carcinoma	White	F	61	1	0.54
49	Infiltrating duct carcinoma	White	F	69	1	2.26
50	Invasive carcinoma	White	F	48	1	0.77
51	Infiltrating duct carcinoma	White	F	75	1	0.94
52	Infiltrating duct carcinoma	White	F	64	1B	1.52
53	Infiltrating duct carcinoma	Black	F	59	1A	1.02
54	Infiltrating duct carcinoma	White	F	56	1	0.64
55	Infiltrating duct carcinoma	White	F	51	1	0.23
56	Infiltrating duct carcinoma	White	F	38	2	1.65
57	Infiltrating duct carcinoma	White	F	69	2	1.53
58	Infiltrating duct carcinoma	Black	F	41	2	0.71
59	Infiltrating duct carcinoma	White	F	39	2	0.76
60	Infiltrating duct carcinoma	White	F	42	2	0.41
61	Infiltrating duct carcinoma	White	F	73	2	0.32
62	Invasive carcinoma, no special type	White	F	75	2A	3.08
63	Invasive carcinoma, no special type	White	F	80	2A	1.14
64	Invasive carcinoma, no special type	White	F	64	2A	2.11
65	Invasive carcinoma, no special type	White	F	72	2A	1.40
66	Invasive carcinoma, no special type	White	F	57	2B	4.23
67	Invasive carcinoma, no special type	White	F	54	2B	1.96
68	Infiltrating duct carcinoma	White	F	34	2B	1.38
69	Infiltrating duct carcinoma	White	F	44	2A	1.38
70	Infiltrating duct carcinoma	White	F	49	2A	1.10
71	Infiltrating duct and lobular carcinoma	White	F	51	3A	1.15
72	Invasive carcinoma, no special type	White	F	62	3A	0.86
73	Infiltrating duct carcinoma	White	F	62	3A	3.02

74	Infiltrating duct carcinoma	White	F	30	3B	1.10
75	Infiltrating duct carcinoma	White	F	79	3	1.43
76	Infiltrating duct carcinoma	White	F	46	3	1.64
77	Infiltrating duct carcinoma	White	F	48	3	0.58
78	Infiltrating duct carcinoma	White	F	62	3	0.82
79	Infiltrating duct carcinoma	Black	F	46	3	1.45
80	Infiltrating duct carcinoma	White	F	62	3	1.11
81	Infiltrating duct carcinoma	White	F	57	3	0.86
82	Infiltrating duct carcinoma	Asian	F	37	3	0.93
83	Infiltrating duct carcinoma	White	F	56	3	1.89
84	Infiltrating duct and lobular carcinoma	White	F	51	3	1.40
85	Infiltrating duct carcinoma	Black	F	42	4	0.75
86	Infiltrating duct carcinoma	White	F	39	4	1.46
87	Infiltrating duct carcinoma	White	F	42	4	0.62
88	Infiltrating duct carcinoma	White	F	48	4	0.65
89	Infiltrating duct carcinoma	White	F	57	4	1.23
90	Infiltrating duct carcinoma	White	F	58	4	0.47
91	Carcinoma	White	F	68	4	1.06
92	Infiltrating duct carcinoma	White	F	65	4	1.13
93	Infiltrating duct carcinoma	White	F	73	4	-0.07
94	Infiltrating duct carcinoma	White	F	77	4	1.48
95	Infiltrating duct carcinoma	White	F	53	4	2.67
96	Infiltrating duct carcinoma	White	F	40	4	0.37
97	Infiltrating duct carcinoma	White	F	53	4	5.76
98	Infiltrating duct carcinoma	White	F	66	4	4.41
99	Infiltrating duct carcinoma	White	F	70	4	0.38
**Test set (5 normal controls and 25 cancer cases)**						
100	Normal control*	White	F	77		-1.09
101	Normal control*	White	F	62		-0.47
102	Normal control*	White	F	48		-0.81
103	Atypical ductal hyperplasia	White	F	50	Benign	0.27
104	Fibrocystic Change	White	F	47	Benign	-0.25
105	Intraductal carcinoma, noninfiltrating, NOS	White	F	55	0	0.57
106	Intraductal carcinoma, noninfiltrating, NOS	White	F	44	0	0.68
107	Intraductal carcinoma, noninfiltrating, NOS	Black	F	76	0	1.26
108	DCIS, NOS	White	F	73	0	0.48
109	DCIS, NOS	White	F	69	0	1.45

110	Infiltrating duct carcinoma, NOS	White	F	51	1	0.83
111	Infiltrating duct carcinoma, NOS	White	F	72	1	1.08
112	Infiltrating duct carcinoma, NOS	White	F	68	1A	0.70
113	Infiltrating duct carcinoma, NOS	White	F	50	1A	0.50
114	Infiltrating duct carcinoma, NOS	White	F	64	1A	0.73
115	Infiltrating duct carcinoma, NOS	White	F	62	2A	1.26
116	Infiltrating duct carcinoma, NOS	White	F	68	2A	0.57
117	Invasive carcinoma, no special type	White	F	68	2A	0.73
118	Invasive carcinoma, no special type	White	F	35	2A	0.91
119	Invasive carcinoma, no special type	White	F	54	2B	0.61
120	Lobular carcinoma, NOS	White	F	78	3C	1.40
121	Lobular carcinoma, NOS	White	F	76	3A	0.59
122	Lobular carcinoma, NOS	White	F	52	3A	1.38
123	Infiltrating duct carcinoma, NOS	Black	F	75	3B	0.52
124	Infiltrating duct and lobular carcinoma	Black	F	59	3A	0.70
125	Infiltrating duct carcinoma, NOS	Native American	F	41	4	0.90
126	Infiltrating duct carcinoma, NOS	White	F	55	4	0.32
127	Invasive carcinoma, no special type	White	F	61	4	0.73
128	Infiltrating duct carcinoma, NOS	White	F	64	4	0.70
129	Invasive carcinoma, no special type	White	F	51	4	0.81

*These normal controls are the same normal controls #85, #86 and #87 in Table S1.
