# Supplementary material for: Exosomal Thomsen–Friedenreich Glycoantigen: A New Liquid Biopsy Biomarker for Lung and Breast Cancer Diagnoses
Source: Cancer Res Commun. 2024 Aug 6;4(8):1933–45. doi: 10.1158/2767-9764.CRC-23-0505 (PMC11302018; doi:10.1158/2767-9764.CRC-23-0505)
Supplement: Figure S1 — Supplementary Figure S1. SPR assay detects exosomal TF-Ag-α for cancer diagnosis. Representative SPR curves for the detection of exosomal TF-Ag-α in serum samples from (a) a Stage II lung cancer patient using a biochip modified with IgG3 negative control antibodies; (b) a Stage 0 breast cancer patient using a biochip modified with IgG3 negative control antibodies, (c) a male normal control using a biochip modified with JAA-F11 antibodies, (d) a male normal control using a biochip modified with IgG3 negative control antibodies, (e) a female normal control using a biochip modified with JAA-F11 antibodies, and (f) a female normal control using a biochip modified with IgG3 negative control antibodies. [file crc-23-0505_figure_s1_supps1.pdf]

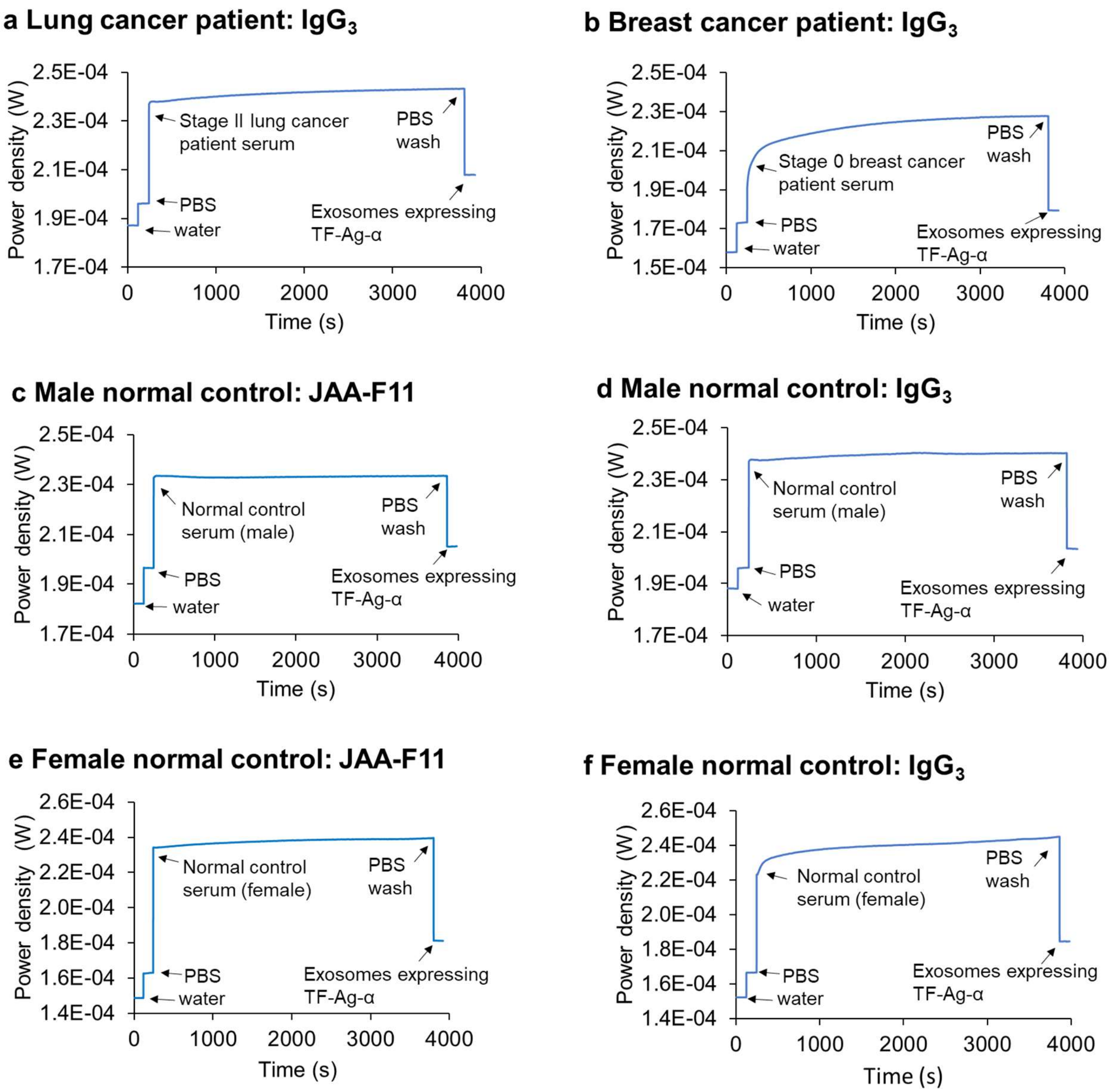

Supplementary Figure S1. SPR assay detects exosomal TF-Ag-α for cancer diagnosis. Representative SPR curves for the detection of exosomal TF-Ag-α in serum samples from (a) a Stage II lung cancer patient using a biochip modified with IgG_3 negative control antibodies; (b) a Stage 0 breast cancer patient using a biochip modified with IgG_3 negative control antibodies, (c) a male normal control using a biochip modified with JAA-F11 antibodies, (d) a male normal control using a biochip modified with IgG_3 negative control antibodies, (e) a female normal control using a biochip modified with with JAA-F11 antibodies, and (f) a female normal control using a biochip modified with IgG_3 negative control antibodies.
