# Supplementary material for: Exosomal Thomsen–Friedenreich Glycoantigen: A New Liquid Biopsy Biomarker for Lung and Breast Cancer Diagnoses
Source: Cancer Res Commun. 2024 Aug 6;4(8):1933–45. doi: 10.1158/2767-9764.CRC-23-0505 (PMC11302018; doi:10.1158/2767-9764.CRC-23-0505)
Supplement: Figure S2 — Supplementary Figure S2. SPR assay demonstrated exosomes carry TF-Ag-α. Exosomes were isolated from cancer patient serum samples using both total exosome isolation kit (from serum) (ThermoFisher) and exoRNeasy midi kit (Qiagen). Significantly higher levels of TF-Ag-α were observed in exosome samples isolated using both kits than the exosome-depleted serum samples. The exosome concentration was 3.29x10^11 exosomes/mL. [file crc-23-0505_figure_s2_supps2.pdf]

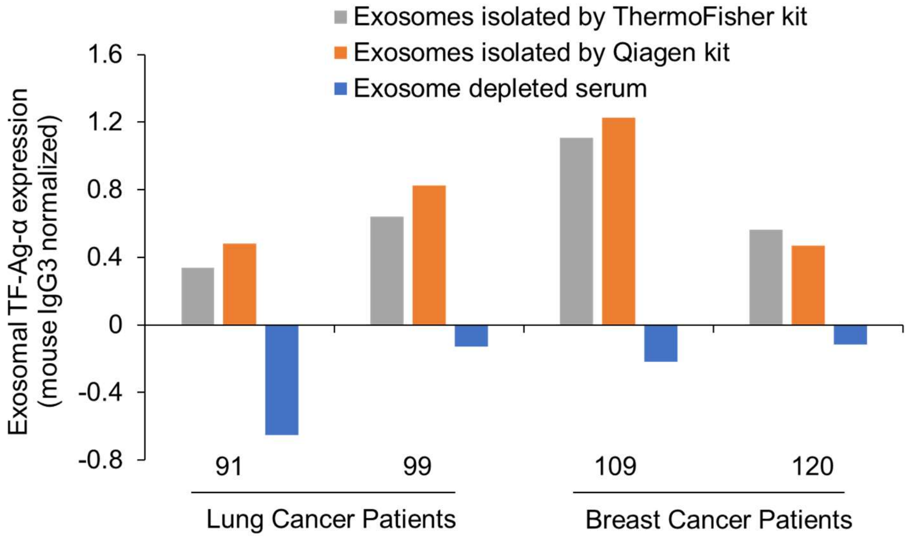

Supplementary Figure S2. SPR assay demonstrated exosomes carry TF-Ag-α. Exosomes were isolated from cancer patient serum samples using both total exosome isolation kit (from serum) (ThermoFisher) and exoRNeasy midi kit (Qiagen). Significantly higher levels of TF-Ag-α were observed in exosome samples isolated using both kits than the exosome-depleted serum samples. The exosome concentration was 3.29×10^{11} exosomes/mL.
