# Supplementary material for: Exosomal Thomsen–Friedenreich Glycoantigen: A New Liquid Biopsy Biomarker for Lung and Breast Cancer Diagnoses
Source: Cancer Res Commun. 2024 Aug 6;4(8):1933–45. doi: 10.1158/2767-9764.CRC-23-0505 (PMC11302018; doi:10.1158/2767-9764.CRC-23-0505)
Supplement: Figure S3 — Supplementary Figure S3. Sensing performance of SPR assay in detecting exosomal TF-Ag-α. Exosomes from A549 NSCLC cells (a) and MDA-MB-231 breast cancer cells (b) were spiked in the serum of a normal control at concentrations of 0 to 10^11 exosomes/mL. The expression of exosomal TF-Ag-α was measured using the SPR assay. For A549 cell-derived exosomes, the LOD was 5×10^9 exosomes/mL and the linear range was from 5×10^9 to 10^11 exosomes/mL. For MDA-MB-231 cell-derived exosomes, the LOD was 10^9 exosomes/mL and the linear range was from 10^9 to 10^11 exosomes/mL. [file crc-23-0505_figure_s3_supps3.pdf]

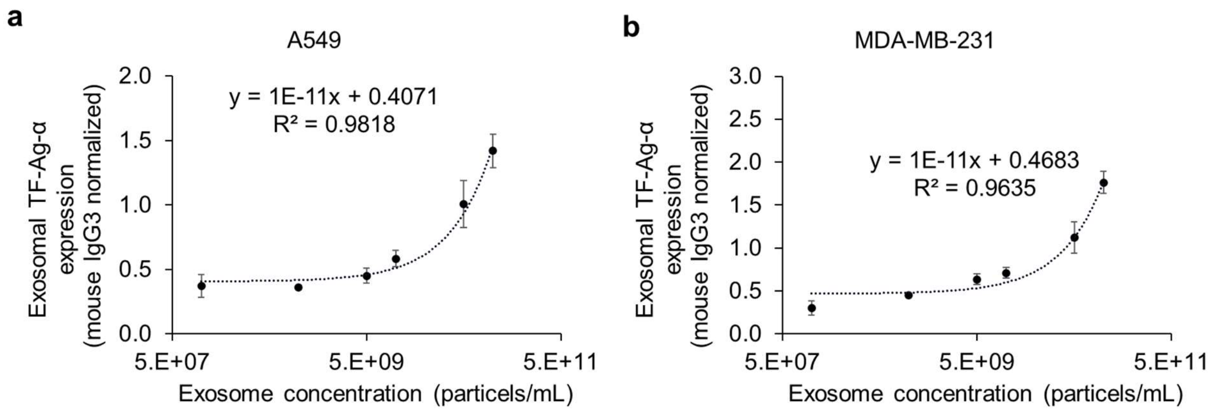

Supplementary Figure S3. Sensing performance of SPR assay in detecting exosomal TF-Ag-α. Exosomes from A549 NSCLC cells (a) and MDA-MB-231 breast cancer cells (b) were spiked in the serum of a normal control at concentrations of 0 to 10^{11} exosomes/mL. The expression of exosomal TF-Ag-α was measured using the SPR assay. For A549 cell-derived exosomes, the LOD was 5×10^9 exosomes/mL and the linear range was from 5×10^9 to 10^{11} exosomes/mL. For MDA-MB-231 cell-derived exosomes, the LOD was 10^9 exosomes/mL and the linear range was from 10^9 to 10^{11} exosomes/mL.
