# Supplementary material for: Exosomal Thomsen–Friedenreich Glycoantigen: A New Liquid Biopsy Biomarker for Lung and Breast Cancer Diagnoses
Source: Cancer Res Commun. 2024 Aug 6;4(8):1933–45. doi: 10.1158/2767-9764.CRC-23-0505 (PMC11302018; doi:10.1158/2767-9764.CRC-23-0505)
Supplement: Supplementary Figure S4 — Repeatability and reproducibility of SPR assay in detecting exosomal TF-Ag-α. The levels of exosomal TF-Ag-α in serum samples from one normal control, one lung cancer patient and one breast cancer patient were measured by operator 1 (a) and operator 2 (b) on 3 different days, 3 replicates per day. The mean, standard deviation, and coefficient of variation (CV) were reported. (c) Pearson correlation of results from operator 1 and operator 2. [file crc-23-0505_supplementary_figure_s4_supps4.pdf]

a Operator 1

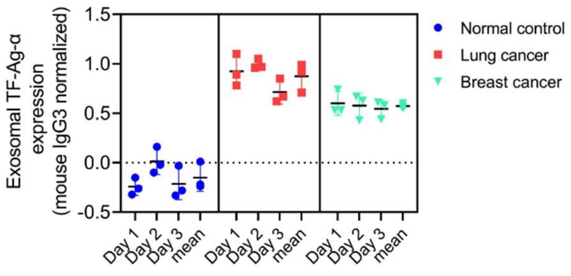

b Operator 2

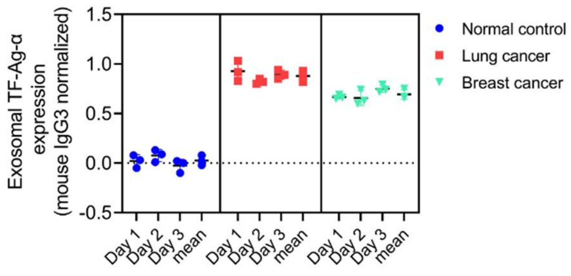

Operator 1:

**Mean**	Day 1	Day 2	Day 3	3-day avg
Normal control	-0.24	0.01	-0.22	-0.15
Lung cancer	0.92	0.99	0.71	0.88
Breast cancer	0.60	0.57	0.55	0.57

**Standard deviation**	Day 1	Day 2	Day 3	3-day avg
Normal control	0.07	0.11	0.13	0.12
Lung cancer	0.13	0.04	0.10	0.12
Breast cancer	0.10	0.11	0.07	0.02

**CV(%)**	Day 1	Day 2	Day 3	3-day avg
Normal control	-29.48%	741.96%	-60.28%	-78.17%
Lung cancer	14.15%	4.34%	13.89%	13.55%
Breast cancer	16.44%	18.46%	13.4%	3.78%

Operator 2:

**Mean**	Day 1	Day 2	Day 3	3-day avg
Normal control	0.02	0.08	-0.02	0.02
Lung cancer	0.93	0.82	0.89	0.88
Breast cancer	0.67	0.66	0.75	0.69

**Standard deviation**	Day 1	Day 2	Day 3	3-day avg
Normal control	0.05	0.05	0.05	0.04
Lung cancer	0.08	0.02	0.04	0.04
Breast cancer	0.02	0.06	0.03	0.04

**CV(%)**	Day 1	Day 2	Day 3	3-day avg
Normal control	235.30%	67.12%	-220.81%	162.26%
Lung cancer	8.57%	2.25%	4.31%	4.84%
Breast cancer	2.79%	9.27%	3.75%	5.93%

c

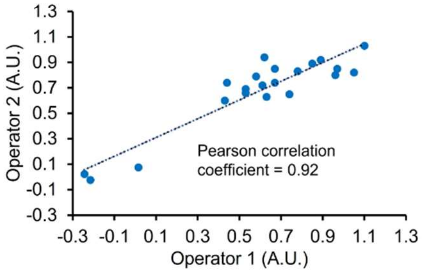

Supplementary Figure S4. Repeatability and reproducibility of SPR assay in detecting exosomal TF-Ag-α. The levels of exosomal TF-Ag-α in serum samples from one normal control, one lung cancer patient and one breast cancer patient were measured by operator 1 (a) and operator 2 (b) on 3 different days, 3 replicates per day. The mean, standard deviation, and coefficient of variation (CV) were reported. (c) Pearson correlation of results from operator 1 and operator 2.
